# High Contrast Far-Field Radiative Thermal Diode

**DOI:** 10.1038/s41598-017-06804-w

**Published:** 2017-07-24

**Authors:** Alok Ghanekar, Gang Xiao, Yi Zheng

**Affiliations:** 1 Department of Mechanical, Industrial and Systems Engineering, University of Rhode Island, Kingston, RI 02881 USA; 2 Department of Physics, Brown University, Providence, RI 02912 USA

## Abstract

We propose a theoretical concept of a far-field radiative thermal rectification device that uses a phase change material to achieve a high degree of asymmetry in radiative heat transfer. The proposed device has a multilayer structure on one side and a blackbody on other side. The multilayer structure consists of transparent thin film of KBr sandwiched between a thin film of VO_2_ and a reflecting layer of gold. When VO_2_ is in its insulating phase, the structure is highly reflective due to the two transparent layers on highly reflective gold. When VO_2_ is in the metallic phase, Fabry-Perot type of resonance occurs and the tri-layer structure acts like a wide-angle antireflection coating achieved by destructive interference of partially reflected waves making it highly absorptive for majority of spectral range of thermal radiation. The proposed structure forms the active part of configuration that acts like a far-field radiative thermal diode. Thermal rectification greater than 11 is obtained for a temperature bias of 20 K, which is the highest rectification ever predicted for far-field radiative diode configurations.

## Introduction

Thermal diode^1^, thermal transistors^2^, thermal memory element^3^ and similar thermal analogues of electronic devices have been topic of theoretical as well as experimental works. While earlier research has been on conduction (phonon) based devices^4–8^, more recent studies have been focusing on radiation (photon) based thermal rectifiers^9–12^. Thermal rectification has numerous applications in thermal management, thermal logic gates^13–15^ and information processing^16^.

Analogous to electrical diode, thermal diode is a rectification device wherein magnitude of heat flux strongly depends on the sign of applied temperature bias. To quantify rectification, we employ the widely used definition of rectification ratio, i.e., *R* = (*Q*_*f*_ − *Q*_*r*_)/*Q*_*r*_ where *Q*_*f*_ and *Q*_*r*_ refer to forward and reverse heat flux, respectively^17^. Alternatively, rectification coefficient can be defined as *η* = (*Q*_*f*_ − *Q*_*r*_)/*max*(*Q*_*r*_, *Q*_*f*_). There are numerous studies pertaining to near-field and far-field thermal radiation based rectification devices that exploit temperature dependent properties of a phase change materials such as vanadium dioxide (VO_2_) and La_0.7_Ca_0.15_Sr_0.15_MnO_3_ (LCSMO)^11, 18, 19^. A number of studies deal with far-field thermal radiation^20, 21^ while several others focus on modulation of radiative heat transfer in the near-field regime^18, 19, 22–26^. Ben-Abdallah and Biehs introduced a VO_2_ based simple far-field radiative thermal diode, while Prod’homme *et al*.^27^, proposed a far-field thermal transistor that uses a VO_2_ base between a blackbody collector and a blackbody emitter. Zhu *et al*.^28^, showed that temperature dependent optical properties of SiC can be used to attain negative differential conductance. Van Zwol *et al*.^22^, proposed that one can take advantage of the phase transition from crystalline to amorphous state in AIST (an alloy of Ag, In, Sb, and Te) driven by a current pulse to obtain a large contrast in heat flux. In far-field limit, rectification is due to the change in emissive properties of a phase change material. In near-field limit, the difference in the coupling strength of polaritons or tunneling of surface waves between structures leads to thermal rectification. In general, it is observed that a higher rectification can be achieved in the near-field regime than in the far-field. However, it is challenging to develop such devices operating on the principle of near-field radiative transfer.

Spectral control has been studied to affect radiative heat transfer in both the far-field as well as near-field. Customization of absorption/emission spectra is often achieved by the use of multilayer thin film structures^29^, nanoparticles^30, 31^, dielectric mixtures^32, 33^, photonic crystals^34, 35^, 1-D/2-D gratings^36^ and metamaterials^37, 38^. Absorbers that utilize Fabry-Perot cavities^39, 40^, Salibury screens^41^ and Jaumann absorbers^42^ and ultra-thin lossy thin films bounded by transparent substrate and superstate^43–45^ have been investigated for decades. Quite notably, Nefzaoui *et al*.^46^, proposed using multilayer structures consisting of thin films (e.g., Si, HDSi and gold) to obtain thermal rectification. Kats *et al*.^47^, have theoretically and experimentally demonstrated that a thin-film of VO_2_ on sapphire shows strong modulation of absorbance upon phase transition, particularly, at wavelength of 11.6 *μ*m. Taylor *et al*.^48^, recently proposed an emitter consisting a dielectric spacer between VO_2_ film and a reflecting substrate to achieve dynamic radiative cooling upon phase transition of VO_2_. Fabry-Perot resonance was achieved at 10 *μ*m wavelength. As discussed later, we show that, by tuning the resonance at right wavelength, maximum rectification can be achieved in the proposed design.

VO_2_ has often been used in thermal rectification devices, because its phase-change from an insulator to a metal can be switched reversibly within a short time (~100 fs)^49^. The common devices use either a bulk VO_2_ solid or its thin-film form. In this work, we present a VO_2_ based far-field thermal rectification device with a simple multilayer structure. We predict a record rectification factor of greater than 11 (*η* > 0.91).

**Figure 1:**
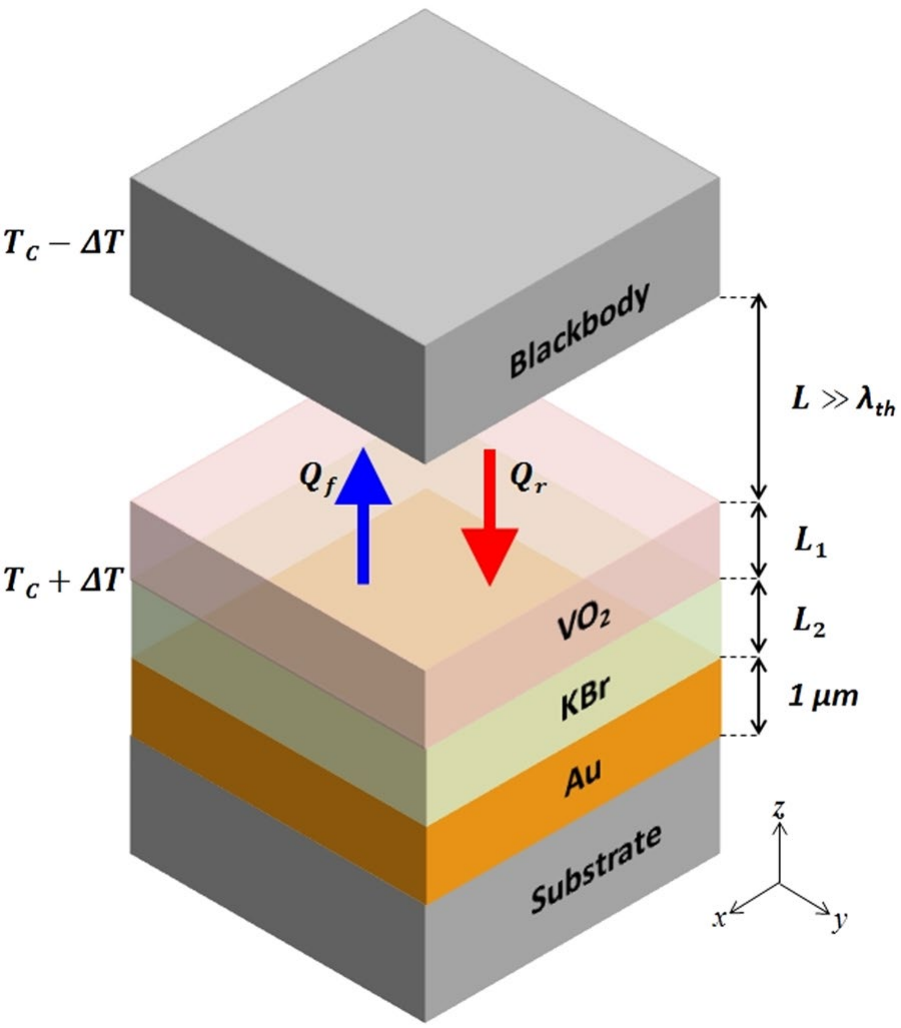
Schematic of a far-field thermal diode with a high rectification ratio. The active component has a tri-layer structure consisting of VO_2_, KBr and gold thin films on a substrate with thicknesses *L*_1_, *L*_2_ and 1 *μ*m, respectively. The passive component is a blackbody. *T*_*c*_ = 341 K is the phase transition temperature of VO_2_.

A typical far-field thermal diode has two planar components separated by a distance much larger than thermal wavelength. The active component is made of a phase-change solid, whereas the passive component stays inert. Figure 1 illustrates the vertical structure of our proposed thermal diode. The active component contains a tri-layer structure consisting of VO_2_, potassium bromide (KBr) and gold thin films on a substrate. Thicknesses of VO_2_ and KBr layers can be tuned to maximize rectification. The thickness of gold layer is fixed at 1 *μ*m to block radiation from the substrate. For a given temperature bias, maximum (far-field) radiative heat transfer would be possible when both sides are blackbodies, while minimum heat transfer would take place when at least one side is a highly reflective mirror. Ideally, the active component should exhibit a transition from blackbody to reflective surface upon the reversal of a temperature bias which induces the phase change. This is exactly our design attempts to achieve. Therefore, the passive component is chosen to be a blackbody. Any material other than a blackbody would not yield the maximum rectification. Structure 1 and 2 are at temperature *T*_1_ = *T*_*C*_ + Δ*T* and *T*_2_ = *T*_*C*_ − Δ*T*, respectively. The mean temperature is chosen to be the phase transition temperature of VO_2_ (*T*_*C*_ = 341 K). When *T*_1_ > *T*_2_ (referred to as forward bias), VO_2_ layer is in its metallic phase; and when *T*_1_ < *T*_2_ (reverse bias), VO_2_ layer becomes insulating with its optical axis aligned along the vertical direction, i.e., z-axis.

Phase transition of VO_2_ is not abrupt^49, 50^ and a complete insulator-metal transition does not occur until 350 K^26^. Rectification ratio depends on temperature bias as the temperature dependence of radiative heat transfer is essentially nonlinear. We calculate rectification values at a minimal temperature bias of 20 K i.e., Δ*T* = ±10 K. Although transition of VO_2_ exhibits a thermal hysteresis of about 8 K as presented in refs 49, 51, the phase transition is reversible. As we are concerned with heat flux values at 10 K above and below the critical temperature of VO_2_, hysteresis behavior is beyond the scope of this study.

## Results and Discussion

A multilayer structure can be designed to attain high absorbance or reflectance based on its dimensions and material properties. Multilayers with constituent thicknesses much smaller than the incident wavelength of light have been studied before^52^. We show that in a VO_2_ based multilayer structure, the dramatic change in the optical property of VO_2_ upon phase-change facilitates an extensive variation in the surface reflectivity.

Concept shown in Fig. 1 has variable dimensions of VO_2_ (*L*_1_) and KBr (*L*_2_) layer. These dimensions were optimized by running Genetic Algorithm to maximize rectification ratio. Matlab’s optimization toolbox was used to run Genetic Algorithm to perform optimization. Default values of population size (50), fitness scaling (rank), crossover fraction (0.8), stopping criteria (100 generations) were selected in the optimization toolbox. No tuning of optimization parameters was required as number of variables was only two. Lower and upper bounds on both *L*_1_ and *L*_2_ were kept at 25 nm and 2 *μ*m, respectively. Optimal dimensions were found to be *L*_1_ = 25 nm and *L*_2_ = 880 nm, both are practical values. Further discussion will be focused on the design with these dimensions.

**Figure 2:**
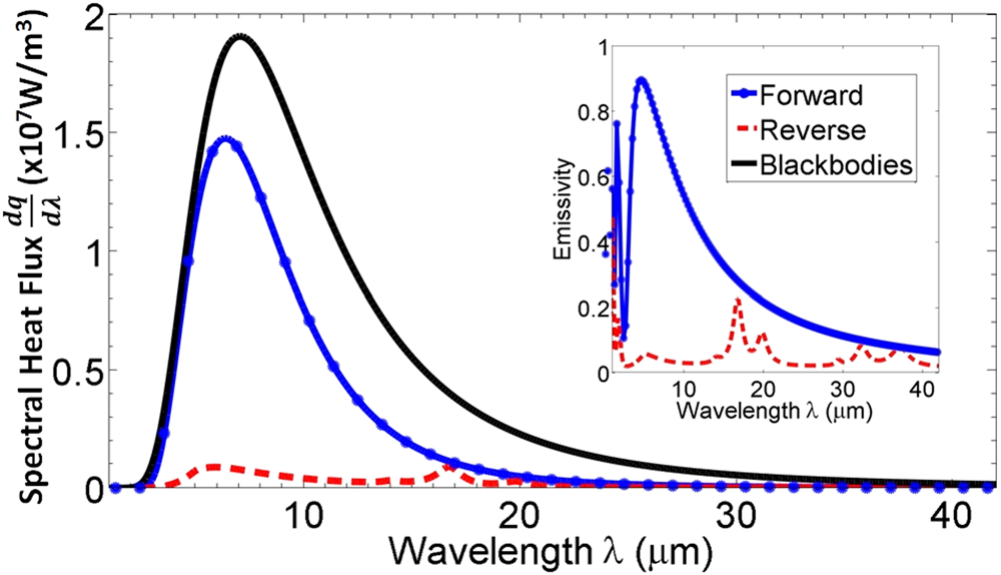
Spectral heat flux across the optimized thermal diode in forward and reverse bias scenarios. Spectral heat flux between blackbodies at temperatures 331 K and 351 K is shown for reference. Inset shows hemispherical emissivity of the active component of the diode for the forward and reverse bias.

**Figure 3:**
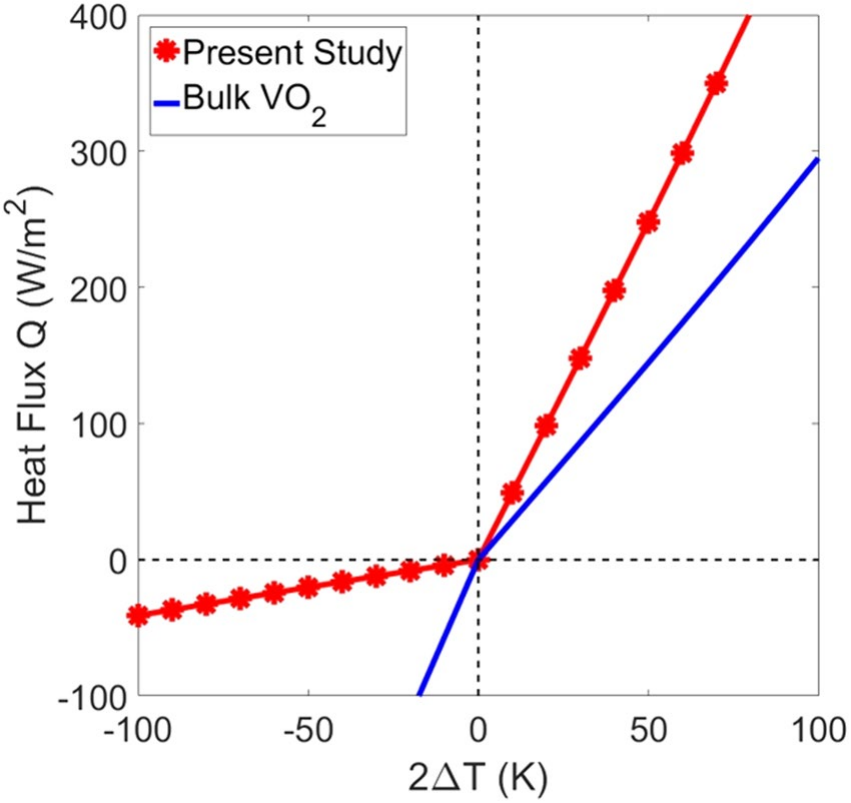
Heat flux plotted against temperature difference for thermal diode with bulk VO_2_ and present structure.

**Figure 4:**
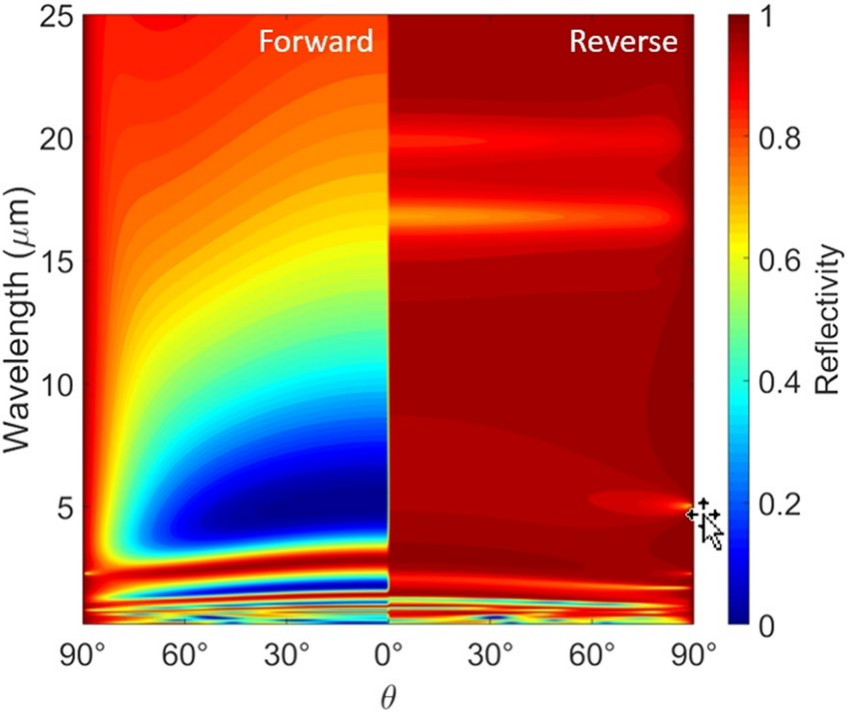
Angle dependent reflectivity of the active component of thermal diode plotted against wavelength and angle of incidence under forward and reverse bias.

Figure 2 shows spectral heat flux (*dq*/*dλ*) of the proposed thermal diode in forward and reverse direction with temperature bias 20 K (Δ*T* = 10 K). Forward heat flux is significantly higher than reverse flux as is clear from Fig. 2. A comparison is shown for heat flux across blackbodies at temperatures 331 K and 351 K, respectively. Inset in Fig. 2 displays angle-averaged emissivity of the active component in both scenarios. When VO_2_ is metallic, the structure on the active component has high emissivity near the thermal wavelength (*λ*_*th*_ = 1.27*ħc*/*k*_*B*_ *T* = 8.5 *μ*m for 341 K). As a significant portion of blackbody radiation falls within this range, this gives rise to a high heat flux in forward bias. However, when VO_2_ is insulating, the structure has very low emissivity in the broad spectrum. The tri-layer structure behaves like a highly reflecting mirror resulting in very low heat flux. Consequently, high contrast in heat flow is achieved leading to a high rectification ratio of 11.3 (*η* = 0.918). In order to highlight the diode-like characteristics, heat flux across the device has been plotted against themeprature difference in Fig. 3. For comparison, simple case of bulk VO_2_ is also shown, it has a rectification coeffcient of *η* = 0.49. Note that, effect of thermal hystersis is not considered here for simplicity. Angle dependent spectral reflectivity of the active component of the thermal diode is plotted in Fig. 4 for the forward and reverse bias cases. When VO_2_ is metallic, the tri-layer structure acts like a wide-angle antireflection coating for wavelengths between 4 *μ*m to 10 *μ*m. The dark spot in Fig. 4 corresponds to Fabry-Perot type of resonance that occurs around *λ* = 4*n*_*KBr*_(*λ*)*L*_2_ = 5.3 *μ*m^47^. High absorption/emission in this wavelength region favors radiative heat transfer as thermal wavelength falls within this range. In reverse bias, the structure is highly reflective in a broad range of wavelengths giving rise to a very low absorption. Note that for thermal wavelength of 8.5 *μ*m, Fabry-Perot resonance occurs (for metallic VO_2_) when thickness of KBr layer is *L*_2_ = *λ*_*th*_/4*n*_*KBr*_(*λ*_*th*_) = 1.4 *μ*m. This configuration however, would not necessarily achieve maximum rectification as the structure may not be purely reflecting when VO_2_ is its insulating phase.

Contrasting reflective properties of the structure are due to constructive and destructive interferences of electromagnetic waves generated by partial reflections at interfaces. As an electromagnetic wave travels through the media, it is partially reflected at each interface leading to multiple reflections from each layer. This causes interference of electromagnetic waves due to each partial reflection. Effective reflection coefficient of the structure is the phasor sum of these reflection coefficients due to (an infinite number of) individual reflections. When VO_2_ is metallic, phasor sum of partial reflections results in destructive interference in the wavelength range of 4 *μ*m to 10 *μ*m. As a result, the structure is highly absorptive in the range. When VO_2_ is insulating, individual reflections add up to a large value making the structure highly reflective for a broad range of the spectrum.

**Figure 5:**
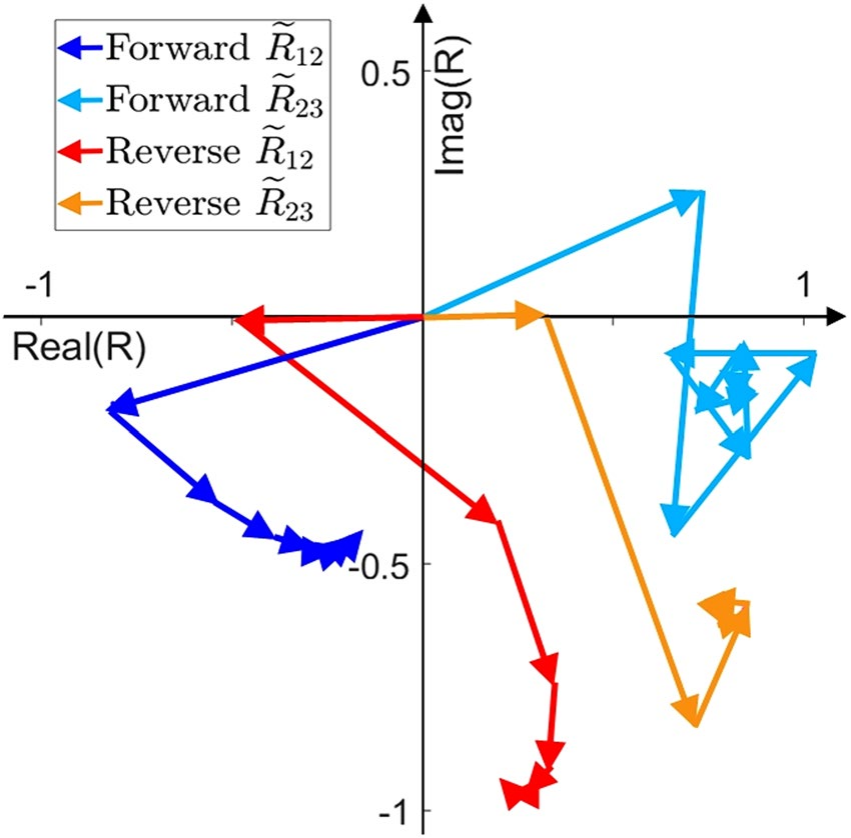
Effective reflection coefficient at air-VO_2_ interface (${\tilde{R}}_{\mathrm{1,2}}$) and VO_2_-KBr interface (${\tilde{R}}_{\mathrm{2,3}}$) as phasor sum of reflection coefficients due to each reflection for TE polarized incident plane wave of wavelength *λ*_*th*_ = 8.5 *μ*m and angle of incidence 10°.

Figure 5 shows phasor diagram of partial reflections at air-VO_2_ interface and VO_2_-KBr interface for TE polarized incident ray of wavelength *λ*_*th*_ = 8.5 *μ*m and angle of incidence 10°. ${\tilde{R}}_{\mathrm{1,2}}$ is the effective reflection coefficient at air-VO_2_ interface and ${\tilde{R}}_{\mathrm{2,3}}$ is the effective reflection coefficient at VO_2_-KBr interface due to multiple reflections within KBr layer. They can be expressed as geometric series whose terms are relative amplitudes of partial waves due to first, second and third reflection and so on. For both metallic as well as insulating VO_2_, the magnitude of ${\tilde{R}}_{\mathrm{2,3}}$, $|{\tilde{R}}_{\mathrm{2,3}}|$, is large. However, when VO_2_ is in metallic phase, each partial reflection results in a phase-shift such that partial waves add up destructively leading to a small value of $|{\tilde{R}}_{\mathrm{1,2}}|$ and low reflectivity, especially in the wavelength range centered around thermal wavelength. On the other hand, in reverse bias (insulating VO_2_) phasors add constructively, giving rise to highly reflective surface properties for a broad range of wavelengths. A similar phenomenon can be observed for TM polarization as well. As KBr is transparent and has a negligible extinction coefficient for most of infrared region, much of the absorption takes place within the VO_2_ layer. Transparent layer of KBr mainly influences the reflective properties by altering the phase of the light propagating through the media. Potentially, any other material transparent to infrared light such as magnesium fluoride or intrinsic silicon can be used in this concept. However, optimal dimensions of such a device might be different.

In summary, we present a VO_2_ based far-field radiative thermal diode structure with a high rectification ratio of 11.3. The active component of the proposed device has a tri-layer structure consisting thin films of VO_2_, KBr and gold. As VO_2_ undergoes phase change around 341 K, reflecting properties of the surface are dramatically changed in the spectral region that contributes to significant amount of thermal radiation. Facilitated by Fabry-Perot type of resonance around 5.3 *μ*m, metallic VO_2_ makes the structure behave like a wide-angle antireflection coating while insulating VO_2_ makes it highly reflecting. As a result, high degree of asymmetry in radiative heat transfer is predicted across the tri-layer structure and a blackbody. Contrasting reflecting properties of the structure can be explained using constructive and destructive interference of partial reflections across the interfaces. We optimized layer thicknesses to maximize rectification. Thermal rectification greater than 11 is predicted for temperature difference of 20 K and it is highest among far-field radiative diodes that have been studied. Possibility of attaining higher rectification could be investigated in future by using alternate transparent materials, thinner films of VO_2_ and/or using more number of alternating VO_2_/dielectric layers. Such devices can find numerous applications such as thermal logic devices and thermal management systems.

## Methods

To calculate heat flux in forward and reverse bias across our far-field thermal diode, we use the well known expression of radiative transfer obtained through dyadic Green’s function formalism^53^. Radiative transfer between two planar objects is given by

1 $${Q}_{1\to 2}({T}_{1},{T}_{2},L)={\int }_{0}^{\infty }\frac{d\omega }{2\pi }[{\rm{\Theta }}(\omega ,{T}_{1})-{\rm{\Theta }}(\omega ,{T}_{2})]{T}_{1\to 2}(\omega ,L)$$

where Θ(*ω*, *T*) = (*ħω*/2)coth(*ħω*/2*k*_*B*_ *T*) is the energy of a harmonic oscillator at frequency *ω* and temperature *T*, *ħ* is the reduced Planck constant, and *k*_*B*_ is the Boltzmann constant. The function *T*_1→2_(*ω*, *L*) corresponds to the spectral transmissivity in radiative transfer between media 1 and 2 with a separation of *L* and is expressed as^53^

2 $${T}_{1\to 2}(\omega ,L)={\int }_{0}^{\omega /c}\frac{{k}_{\rho }d{k}_{\rho }}{2\pi }\sum _{\begin{array}{c}\mu =TE,\\ TM\end{array}}\frac{(1-{|{\tilde{R}}_{h1}^{(\mu )}|}^{2})(1-{|{\tilde{R}}_{h2}^{(\mu )}|}^{2})}{{|1-{\tilde{R}}_{h1}^{(\mu )}{\tilde{R}}_{h2}^{(\mu )}{e}^{2j{k}_{hz}L}|}^{2}}$$

where ${\tilde{R}}_{h1}^{(\mu )}$ and ${\tilde{R}}_{h2}^{(\mu )}$ are polarized effective reflection coefficients of the two half spaces (calculated in the absence of other half space), *μ* = *TE* (or *TM*) refers to transverse electric (or magnetic) polarization and *k*_*hz*_ is the *z*-component of wavevector in vacuum. Here, *j* is the imaginary unit. For a structure having *N*-layer media having (*N* − 1) interfaces, by solving the boundary conditions at the interfaces, one can obtain the expression for the generalized reflection coefficient at the interface between regions *i* and *i* + 1^54^,

3 $${\tilde{R}}_{i,i+1}^{(\mu )}=\frac{{R}_{i,i+1}^{(\mu )}+{\tilde{R}}_{i+\mathrm{1,}i+2}^{(\mu )}{e}^{2j{k}_{i+\mathrm{1,}z}({d}_{i+1}-{d}_{i})}}{1+{R}_{i,i+1}^{(\mu )}{\tilde{R}}_{i+\mathrm{1,}i+2}^{(\mu )}{e}^{2j{k}_{i+\mathrm{1,}z}({d}_{i+1}-{d}_{i})}}$$

where ${R}_{i,i+1}^{(\mu )}$ is the Fresnel reflection coefficient at the interface between the layers *i* and *i* + 1, and ${\tilde{R}}_{i+\mathrm{1,}i+2}^{(\mu )}$ is the generalized reflection coefficient at the interface between the layers *i* + 1 and *i* + 2, *z* = −*d*_*i*_ is the location of the *i*th interface. ${k}_{i,z}=\sqrt{{\varepsilon }_{i}(\omega ){\omega }^{2}/{c}^{2}-{k}_{\rho }^{2}}$ is the normal *z*-component of the wave vector in medium *i*, wherein *ε*_*i*_(*ω*) is the relative permittivity of the medium *i* as a function of angular frequency *ω*, *c* is the speed of light in vacuum and *k*_*ρ*_ is the magnitude of the in-plane wave vector. With ${\tilde{R}}_{N,N+1}^{(\mu )}=0$, the above equation provides a recursive relation to calculate the reflection coefficients ${\tilde{R}}_{i,i+1}^{(\mu )}$ in all regions. Note that Eq. 2 has only one integral corresponding to propagating waves. The terms due to evanescent waves are ignored as separation between the two half spaces is much larger than the thermal wavelength ($L\gg {\lambda }_{th}$). The hemispherical emissivity of the active component can be expressed as^32^

4 $$e(\omega )=\frac{{c}^{2}}{{\omega }^{2}}{\int }_{0}^{\omega /c}d{k}_{\rho }{k}_{\rho }\sum _{\mu =s,p}(1-{|{\tilde{R}}_{h}^{(\mu )}|}^{2})$$

Note that the term for tranmissivity has been omitted as a layer of gold makes the structure opaque.

Insulating VO _2_ (below 341 K) is anisotropic. In a plane (*x* − *y* plane in Fig. 1) perpendicular to optical axis known as the ordinary mode, its dielectric function is *ε*_*O*_ and it is *ε*_*E*_ along the optical axis (extraordinary mode). Both *ε*_*O*_ and *ε*_*E*_ can be calculated using the classical oscillator formula $\varepsilon (\omega )={\varepsilon }_{\infty }+{\sum }_{i=1}^{N}\frac{{S}_{i}{\omega }_{i}^{2}}{{\omega }_{i}^{2}-j{\gamma }_{i}\omega -{\omega }^{2}}$. Values of high-frequency constant *ε*_∞_, phonon frequency *ω*_*i*_, scattering rate *γ*_*i*_ and oscillator strength *S*_*i*_ are taken from ref. 55. There are eight phonon modes for ordinary and nine phonon modes for extraordinary dielectric function. In the metallic state, VO_2_ is isotropic and Drude model^55^ is used to describe the dielectric function i.e., $\varepsilon (\omega )=\frac{-{\omega }_{p}^{2}{\varepsilon }_{\infty }}{{\omega }^{2}-j\omega {\rm{\Gamma }}}$. Refractive indices of KBr are taken from ref. 56, while dielectric properties of gold can be found in ref. 57. Blackbody is assumed to have a constant dielectric function *ε* = 1 + 0.001*j*.
